# CXCR4, CXCR5 and CD44 May Be Involved in Homing of Lymphoma Cells into the Eye in a Patient Derived Xenograft Homing Mouse Model for Primary Vitreoretinal Lymphoma

**DOI:** 10.3390/ijms231911757

**Published:** 2022-10-04

**Authors:** Neele Babst, Lisa K. Isbell, Felix Rommel, Aysegul Tura, Mahdy Ranjbar, Salvatore Grisanti, Cordula Tschuch, Julia Schueler, Soroush Doostkam, Peter C. Reinacher, Justus Duyster, Vinodh Kakkassery, Nikolas von Bubnoff

**Affiliations:** 1 Department of Ophthalmology, University of Lübeck, Ratzeburger Allee 160, 23538 Lübeck, Germany; 2 Department of Medicine I, Medical Center—University of Freiburg, Faculty of Medicine, 79106 Freiburg, Germany; 3 Charles River Discovery Research Services GmbH, 79108 Freiburg, Germany; 4 Institute for Neuropathology, Medical Center—University of Freiburg, Faculty of Medicine, 79106 Freiburg, Germany; 5 Department of Stereotactic and Functional Neurosurgery, Medical Center—University of Freiburg, Faculty of Medicine, 79106 Freiburg, Germany; 6 Fraunhofer Institute for Laser Technology (ILT), 52074 Aachen, Germany; 7 German Cancer Consortium (DKTK), Freiburg, Germany and German Cancer Research Center (DKFZ), 69120 Heidelberg, Germany; 8 Department of Hematology and Oncology, Medical Center, University Hospital Schleswig-Holstein, Campus Lübeck, Ratzeburger Allee 160, 23538 Lübeck, Germany

**Keywords:** primary vitreoretinal lymphoma, tropism, patient-derived xenograft mouse model, homing receptors, homing

## Abstract

Background: Primary vitreoretinal lymphoma (PVRL), a rare malignancy of the eye, is strongly related to primary central nervous system lymphoma (PCNSL). We hypothesized that lymphoma cells disseminate to the CNS and eye tissue via distinct homing receptors. The objective of this study was to test expression of CXCR4, CXCR5, CXCR7 and CD44 homing receptors on CD20 positive B-lymphoma cells on enucleated eyes using a PCNSL xenograft mouse model. Methods: We used indirect immunofluorescence double staining for CD20/CXCR4, CD20/CXCR5, CD20/CXCR7 and CD20/CD44 on enucleated eyes of a PCNSL xenograft mouse model with PVRL phenotype (PCNSL group) in comparison to a secondary CNS lymphoma xenograft mouse model (SCNSL group). Lymphoma infiltration was evaluated with an immunoreactive score (IRS). Results: 11/13 paired eyes of the PCNSL but none of the SCNSL group were infiltrated by CD20-positive cells. Particularly the choroid and to a lesser extent the retina of the PCNSL group were infiltrated by CD20+/CXCR4+, CD20+/CXCR5+, few CD20+/CD44+ but no CD20+/CXCR7+ cells. Expression of CXCR4 (*p* = 0.0205), CXCR5 (*p* = 0.0004) and CD44 (*p* < 0.0001) was significantly increased in the PCNSL compared to the SCNSL group. Conclusions: CD20+ PCNSL lymphoma cells infiltrating the eye co-express distinct homing receptors such as CXCR4 and CXCR5 in a PVRL homing mouse model. These receptors may be involved in PVRL homing into the eye.

## 1. Introduction

Primary vitreoretinal lymphoma (PVRL) is a severe cancer of the eye, which infiltrates the vitreous and retina. It is a rare non-Hodgkin lymphoma (NHL) with an estimated incidence of approximately 0.05/100.000 [1,2,3]. However, PVRL is the most common primary intraocular lymphoma [2]. PVRL is a disease of the elderly with a mean age of 60 years. Both sexes are equally affected [1]. Few risk factors for the development of PVRL are known. The lymphoma is associated with immunosuppression due to HIV- or EBV-infection [4,5]. Nevertheless, an increasing incidence of PVRL has been noted for immunocompetent patients [6]. In 95% of cases PVRL is diagnosed to be of the diffuse large B-cell-lymphoma (DLBCL) type [3]. PVRL is strongly related to primary central nervous system lymphoma (PCNSL) and is considered to be a subgroup of this lymphoma entity. PCNSL represent 4–6% of all brain tumours [2,7]. In patients diagnosed with PCNSL, 20% eventually develop intraocular involvement. Conversely, 80% of patients with PVRL develop lymphomatous CNS infiltration during the course of the disease [8,9,10]. Due to the CNS manifestation, PVRL is associated with a poor prognosis leading to a 1 year overall survival rate of 25–40%. The median overall survival rate for isolated PVRL is 58 months [1].

The pathogenesis of PVRL is not well understood. How and why lymphoma cells disseminate into the eyes and CNS, both considered immune privileged organs, is unclear. [11,12,13]. Various hypotheses are discussed [12,14,15]. It seems most likely that lymphoma cells arise outside of the CNS in the germinal center of lymph nodes and afterwards disseminate via distinct homing receptors to the CNS and eyes [12,16]. This process of directed movement of lymphocytes or lymphoma cells is called homing [17,18,19].

In general, it has been hypothesized also for PVRL, that lymphoma cell homing can be mediated by several receptor-ligand interactions. The process of homing can be divided into four steps called rolling, activation, adhesion, and diapedesis through the endothelium [20,21]. After leaving the bloodstream, the further migration of lymphoma cells in the tissue occurs either randomly or based on concentration gradients of chemokines. The concentration of chemokines is highest at their place of production—for example, in the eye [21]. Lymphoma cells then may infiltrate the vitreous and retina, leading to PVRL. The chemokine-receptors C-X-C-motif receptor 4 (CXCR4), C-X-C-motif receptor 5 (CXCR5) and C-X-C-motif receptor 7 (CXCR7) as well as the so-called homing-receptor cluster of differentiation 44 (CD44) and their ligands C-X-C-motif ligand 12 (CXCL12), C-X-C-motif ligand 13 (CXCL13) and hyaluronic acid (HA) are of particular interest for homing.

CXCR4 and its ligand CXCL12 are ubiquitously expressed; e.g., CXCR4 is expressed by hemopoietic and epithelial stem cells as well as by the endothelium and retinal pigment epithelium (RPE) within the eye [22,23]. However, they are also found to be overexpressed in several types of cancer such as B-cell non-Hodgkin-lymphoma [23,24,25,26]. Both receptor and ligand are overexpressed due to certain stimuli like hypoxia and injury. Binding of CXCL12 to CXCR4 induces chemotaxis as well as angiogenesis and cell proliferation. CXCL12 can also bind to CXCR7, which directly inhibits the CXCR4 receptor. It has been shown that CXCR7+ lymphoma cells compared to CXCR7- lymphoma cells display increased homing to the brain [24,26,27].

CXCR5 is more specific for B- and T-lymphocytes [28]. Its ligand CXCL13 is expressed by follicular dendritic cells in the spleen and other secondary lymphatic organs [25,29]. Physiologically, the CXCR5-CXCL13-axis contributes to the normal structure of lymph nodes. CXCL13 acts as chemotactic agent and thereby attracts CXCR5-positive B-cells into the follicle [30].

The so-called homing-receptor CD44 and its ligand HA are both ubiquitously expressed [31,32,33,34]. Binding of HA to CD44 can induce cell migration and therefore contribute to dissemination and metastasis [35]. Together, activation of chemokine and homing receptors leads to chemotaxis and cell migration via several signaling pathways.

We recently established a novel patient derived xenograft (PDX) PCNSL mouse model with PVRL phenotype, showing CD20 positive lymphoma cells in the retina of the PCNSL PDX model (unpublished, manuscript under review [36]). We hypothesized that CXCR4, CXCR5, CXCR7 and CD44 are expressed on CD20-positive lymphoma cells in the eyes of this PCNSL PDX model. PCNSL and SCNSL PDX models were established via intracerebral (i.c.) implantation of patient stereotactic CNS biopsies and then implanted into the spleen of recipient mice. Intrasplenic (i.s.) transplanted mice developed CNS lymphoma manifestations and in case of PCNSL retinal infiltration, creating a PCNSL and PVRL homing model. To our knowledge, this is the first homing model for PCNSL and PVRL established so far and created the opportunity for the present study (unpublished manuscript under review [36]). Therefore, we have chosen to evaluate the homing receptors CXCR4 and CXCR5 already known to play a role in PVRL pathogenesis as well as CXCR7 and CD44, that have not been analyzed in PVRL so far. The objective of this study was to test expression of the homing receptors CXCR4, CXCR5, CXCR7 and CD44 on CD20 positive B-lymphoma cells in the eyes using a newly established PDX PCNSL respectively PDX SCNSL mouse model.

## 2. Results

### 2.1. CD20-Positive Lymphoma Cells Are Mostly Found in the Choroid in the PCNSL Group

**Figure 1:**
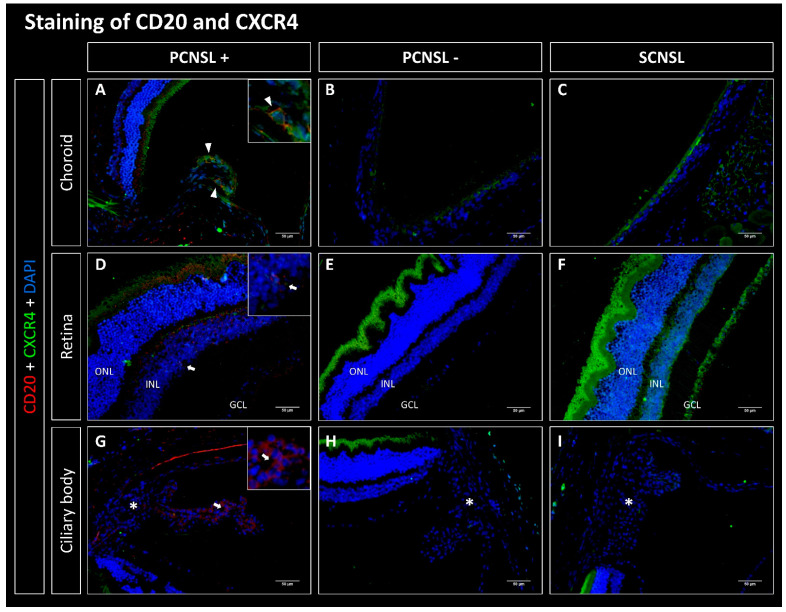
Fluorescence microscopy of CD20 (red) and CXCR4 (green) double staining of the eyes in the PDX PCNSL vs. PDX SCNSL group. (**A**–**C**) show recordings of the choroid, (**D**–**F**) of the retina and (**G**–**I**) of the ciliary body (marked with *). (**A**) shows the choroid infiltrated with CD20-positive primary CNS lymphoma cells (PCNSL+), which additionally express CXCR4 (triangles). (**D**,**G**) show retina and ciliary body infiltrated with CD20-positive cells (PCNSL+) without co-expression. Neither CD20- nor CXCR4-positive cells were found in the PCNSL negative (PCNSL-) group (**B**,**E**,**H**) and in the SCNSL group (**C**,**F**,**I**). ONL = outer nuclear layer, INL = inner nuclear layer, GCL = ganglion cell layer; scale bar 50 µm.

**Figure 2:**
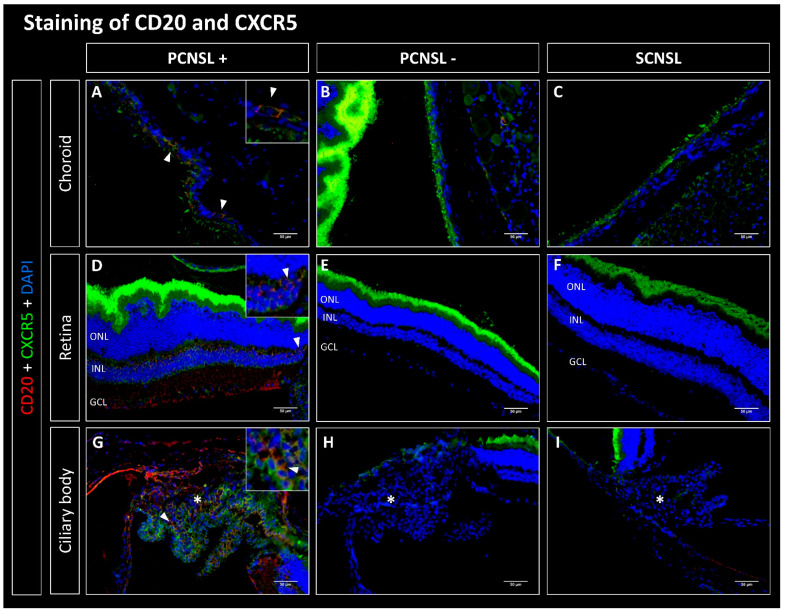
Fluorescence microscopy of CD20 (red) and CXCR5 (green) double staining of the eyes in the PDX PCNSL vs. PDX SCNSL group. (**A**–**C**) show recordings of the choroid, (**D**–**F**) of the retina and (**G**–**I**) of the ciliary body (marked with *). (**A**,**D**,**G**) show the choroid, retina, and ciliary body infiltrated with CD20-positive primary CNS lymphoma cells (PCNSL+), which additionally express CXCR5 (triangles). Neither CD20- nor CXCR5-positive cells were found in the PCNSL negative (PCNSL-) group (**B**,**E**,**H**) and in the SCNSL group (**C**,**F**,**I**). ONL = outer nuclear layer, INL = inner nuclear layer, GCL = ganglion cell layer; scale bar 50 µm.

**Figure 3:**
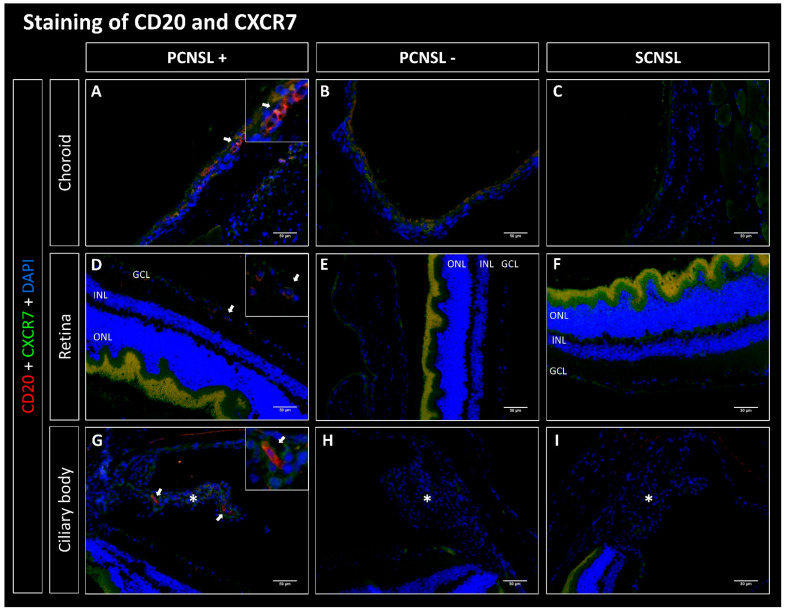
Fluorescence microscopy of CD20 (red) and CXCR7 (green) double staining of the eyes in the PDX PCNSL vs. PDX SCNSL group. (**A**–**C**) show recordings of the choroid, (**D**–**F**) of the retina and (**G**–**I**) of the ciliary body (marked with *). (**A**,**D**,**G**) show the choroids, retinas, and ciliary bodies infiltrated with CD20-positive primary CNS lymphoma cells (PCNSL+) (arrows). Hardly any CXCR7-positive staining was found. No CD20+ cells were found in the PCNSL negative (PCNSL-) group (**B**,**E**,**H**) and in the SCNSL group (**C**,**E**,**I**). In the PCNSL- and SCNSL groups, CXCR7 stained faintly in the choroids (**B**,**C**), weakly to moderately in the retinas (**E**,**F**) and moderately in the ciliary body (**H**,**I**). ONL = outer nuclear layer, INL = inner nuclear layer, GCL = ganglion cell layer; scale bar 50 µm.

**Figure 4:**
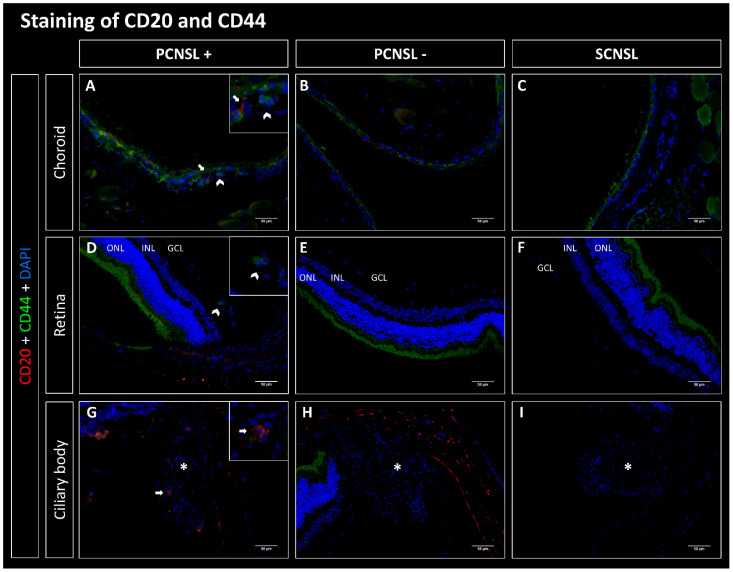
Fluorescence microscopy of CD20 (red) and CD44 (green) double staining of the eyes in the PDX PCNSL vs. PDX SCNSL group. (**A**–**C**) show recordings of the choroid, (**D**–**F**) of the retina and (**G**–**I**) of the ciliary body (marked with *). (**A**,**D**,**G**) show the choroids, retinas, and ciliary bodies infiltrated with CD20-positive primary CNS lymphoma cells (PCNSL+) (arrows). (**A**) shows CD44+ cells (chevron arrow), which are often located in capillaries near CD20-positive lymphoma cells. CD44+ cells were also seen in capillaries in the retina (**D**). Fewer CD44+ cells were found in the ciliary body. In the PCNSL negative (PCNSL-) group (**B**,**E**,**H**) and in the SCNSL group (**C**,**F**,**I**) no CD20+ and few CD44+ cells were found in the choroid, retina and ciliary body. ONL = outer nuclear layer, INL = inner nuclear layer, GCL = ganglion cell layer; scale bar 50 µm.

Lymphoma cells from the established SCNSL PDX model without PVRL (SCNSL group) as well as from the PCNSL PDX model with PVRL phenotype (PCNSL group) were implanted i.s. into 10 and 13 recipient mice, respectively. The evaluation of staining for human CD20 showed that 0/10 paired eyes of the SCNSL group were infiltrated with CD20-positive lymphoma cells (Figure 1, Figure 2, Figure 3 and Figure 4). Within the PCNSL group, 11/13 paired eyes showed positive staining for CD20, whereas 2/13 paired eyes were not infiltrated with CD20-positive lymphoma cells. The lymphoma cells were mostly localized in the choroid, showing positive staining in 11/13 paired eyes. The retina was infiltrated in 7/13 cases. Infiltration with few lymphoma cells was also observed in the sub-retinal and sub-RPE space. In 4/13 paired eyes CD20 positive cells were found in the ciliary body.

### 2.2. CD20-Positive Lymphoma Cells Co-Express CXCR4, CXCR5 and CD44 but Not CXCR7

We next evaluated co-expression of CD20 with CXCR4, CXCR5, CD44 and CXCR7. No co-expressing cells were found in the SCNSL group (Figure 1, Figure 2, Figure 3 and Figure 4). Within the PCNSL group co-expressing cells were predominantly found in the choroid (Figure 1, Figure 2, Figure 3 and Figure 4). These cells primarily co-expressed CD20/CXCR4 (Figure 1) and CD20/CXCR5 (Figure 2). CD20/CD44-positive cells were found sporadically (Figure 4). Only a few cells were found to co-express CD20 and CXCR7 (Figure 3). The same pattern was found in sections of the retina, even though fewer co-expressing cells were detected (Figure 1, Figure 2, Figure 3 and Figure 4). The ciliary body showed little positive staining for CD20 (Figure 1, Figure 2, Figure 3 and Figure 4). However, some of these cells co-expressed CXCR5 (Figure 2).

### 2.3. The CXCR5 Receptor Was Most Frequently Co-Expressed among All Examined Homing-Receptors

Analysis of the manually determined proportion of co-expressing cells out of all CD20+ cells identified CXCR5 as the most frequently co-expressed receptor (Supplementary Materials Figure S1).

#### 2.3.1. Choroid

Across all PCNSL eyes, 32/91 (35%) of the CD20+ cells in the choroid showed co-expression with CXCR5. Analysis of CD20 and CXCR4 staining demonstrated that 21/138 cells (15%) of CD20-positive cells co-expressed CXCR4. The staining of CD20 + CXCR7 and CD20 + CD44 revealed few co-expressing cells. Of 152 CD20-positive cells, three (2%) also showed expression of CXCR7 and of 132 CD20-positive cells, ten (8%) also expressed CD44 (Supplementary Materials Figure S1).

#### 2.3.2. Retina

In the retina, 7/21 (33%) CD20-positive cells revealed co-staining of CXCR5. CXCR7 was co-expressed in 2/30 (7%) of cases. 3/18 (18%) CD20-positive cells co-expressed CD44. CXCR4 was not co-expressed in the retina. Overall, a lower lymphoma cell infiltration was observed in the retina compared to the choroid (Supplementary Materials Figure S1).

#### 2.3.3. Ciliary Body

In the ciliary bodies of the PCNSL group, 1/4 (25%) of the CD20-positive cells also showed co-expression with CXCR5. 2/9 (22%) of cells showed co-expression with CXCR4. CXCR7 was co-expressed in 4/19 (21%) of CD20-positive cells. No CD20-positive cells showed additional staining for CD44. We counted significantly lower infiltration with CD20-positive cells in the ciliary body compared to the retina (Supplementary Materials Figure S1).

### 2.4. CD20, CXCR4, CXCR5 and CD44 Are Expressed Significantly Higher in the PCNSL-Group Compared to the SCNSL-Group

**Figure 5:**
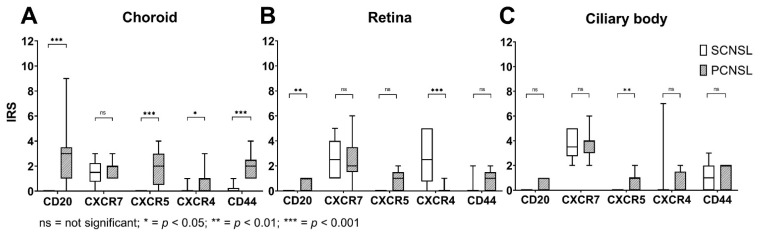
Box Plots of IRS values of CD20, CXCR7, CXCR5, CXCR4, and CD44 of the choroid, the retina, and the ciliary body in the PCNSL vs. SCNSL group. (**A**) Box plots of IRS values in the choroid of the PCNSL vs. SCNSL group. The IRS was significantly higher for CD20 (marked with asteriks *** *p* < 0.001), CXCR5 (marked with asterisks *** *p* < 0.001), CXCR4 (marked with asterisks * *p* < 0.05) and CD44 (marked with asterisks *** *p* < 0.001) in the PCNSL compared to the SCNSL group. (**B**) Box plots of IRS values in the retina of the PCNSL vs. SCNSL group. The IRS was significantly higher for CD20 (marked with asterisks ** *p* < 0.01) and CXCR4 (marked with asterisks *** *p* < 0.001) in the PCNSL compared to the SCNSL group. (**C**) Box plots of IRS values in the ciliary body of the PCNSL vs. SCNSL group. The IRS was significantly higher for CXCR5 (marked with asterisks ** *p*-value < 0.01) in the PCNSL compared to the SCNSL group. ns = not significant.

We next used an immunoreactive score (IRS) to quantify expression of CD20, CXCR4, CXCR5 and CD44 in the eyes in the PCNSL group in comparison to the SCNSL group (Figure 5). To determine the IRS, staining intensity and the percentage of positive cells were scored. We used this score as quality control for our immunoreactive double stainings as well as to evaluate overall expression levels of all cells in the eyes and not only of CD20 positive cells.

#### 2.4.1. Choroid

In the SCNSL group, no CD20-positive cells were found, resulting in an IRS of 0. In contrast, the expression of CD20 in the PCNSL group showed a wide range of variation of IRS values from 0 to 9. The median IRS was 3, indicating that human CD20 positive lymphoma cells were exclusively detected in the PCNSL model. In comparison to the SCNSL group, CXCR5 (*p* = 0.0004), CXCR4 (*p* = 0.0205) and CD44 (*p* < 0.0001) were significantly higher expressed in the PCNSL group. CXCR7 showed weak expression in both groups, with an IRS median of 2 with no differences between the two groups (*p* = 0.7408) (Figure 5).

#### 2.4.2. Retina

Overall, the CD20 IRS values indicated that there were few weakly CD20 positive lymphoma cells in the retina. Again, positive staining for CD20 was only present in the PCNSL group. CXCR4 was moderately expressed in the retina; expression was significantly higher in the SCNSL group than in the PCNSL group (*p* = 0.0008). CXCR5 (*p* = 0.9706) and CD44 (0.0594) also showed a weak expression pattern in both the SCNSL and PCNSL groups and did not differ significantly. CXCR7 (*p* = 0.9706) showed weak to moderate expression in both groups with no significant difference (Figure 5).

#### 2.4.3. Ciliary Body

The CD20 IRS values revealed few CD20-positive lymphoma cells in the ciliary body in the PCNSL group and no CD20-positive cells in the SCNSL group. CXCR5 showed a weak expression pattern in the PCNSL group, being significantly higher than in the SCNSL group (*p* = 0.0075). The receptors CXCR4 (*p* = 0.1573), CXCR7 (*p* = 0.9355) and CD44 (*p* = 0.6809) showed no significant differences between the two groups (Figure 5).

## 3. Discussion

In this study we investigated a novel CNS lymphoma PDX mouse model for a PVRL phenotype. We compared the eyes of a lymphoma PDX model derived from a PCNSL versus SCNSL patient stereotactic CNS biopsy regarding infiltration of human CD20 positive lymphoma cells and immunohistochemical expression of the homing receptors CXCR4, CXCR5, CXCR7 and CD44.

### 3.1. CD20+ Cell Infiltration

Lymphoma cell infiltration of the eye was demonstrated by several researchers using orthotopic mouse models for PVRL. Touitou et al. (2007) and Ben Abdelwahed et al. (2013) both worked with BALBc mice that were injected intravitreally with murine B-lymphoma cells. In both models, the vitreous and the retina were infiltrated with B-lymphoma cells. Additionally, Touitou et al. described that the anterior chamber, the iris, the ciliary body, and the choroid were also infiltrated [37,38]. Li et al. (2006) and Mineo et al. (2008) established xenograft models, in which lymphoma cells were injected intravitreally in SCID or C3H/HeN mice. They obtained comparable results. Mineo et al. were also able to show that B-lymphoma cells can infiltrate the subretinal space, the anterior chamber, and the conjunctiva [39,40].

In our PDX mouse model, we found eye infiltration after heterotopic (intrasplenic) implantation of human PCNSL xenografts, supporting the concept of homing of CNSL cells to the brain and eyes. Here, CD20-positive lymphoma cells were found primarily in the choroid, but the retina and ciliary body were also infiltrated (Figure 1, Figure 2, Figure 3 and Figure 4). Even though an infiltration of the choroid cannot be seen clinically, the choroid may function as a point of entry for lymphoma cells into the retina and is therefore an important anatomical structure to examine in PVRL. Interestingly, we found no infiltration in the vitreous, the anterior chamber, the iris, or the conjunctiva. These slight differences may result from the different methodology used for this mouse model. In this PDX model, DLBCL lymphoma xenograft cells from established PCNSL or SCNSL PDX were injected heterotopically into the spleen of NSG/NOG mice (unpublished manuscript under review [36]). In the case of PCNSL, this led to retinal infiltration, resulting in a homing model for PVRL. Our homing model therefore contrasts with other PVRL mouse models, in which lymphoma cells were injected directly intravitreally, i.e., orthotopically. We were able to show that 11/13 paired eyes of the PCNSL model but none of the paired eyes of the SCNSL model were infiltrated with CD20-positive lymphoma cells, confirming the PVRL phenotype.

### 3.2. Co-Expression of CD20 and Homing Receptors

#### 3.2.1. CD20/CXCR4 Co-Expression

Studies showed CXCR4 and its ligand CXCL12 to be expressed by lymphoma cells in PVRL, PCNSL and primary testicular lymphoma (PTL). Expression of CXCL12 was also found in the retina, the RPE, and cerebral vascular endothelium [39,41,42,43,44,45,46,47,48]. In PTL, expression of CXCR4 was associated with a poor prognosis [47].

In this study, we detected co-expression of CD20 and CXCR4 on lymphoma cells in the PCNSL group but not in the SCNSL group. Thus, our model recapitulates results of previous studies in primary lymphoma samples. When we evaluated the overall expression of CXCR4 in both groups using the IRS, CXCR4 was significantly more highly expressed in the retina in the SCNSL group, contrary to our expectations. However, CXCR4 can be expressed in healthy retina by photoreceptor cells, by the RPE as well as by endothelial cells [22]. In summary, CXCR4 was expressed by CD20-positive lymphoma cells in our PCNSL/PVRL model. Thus, the CXCR4-CXCL12 interaction could mediate the chemotaxis of CD20/CXCR4-positive lymphoma cells in PVRL [41,42].

#### 3.2.2. CD20/CXCR5 Co-Expression

It was shown that CXCR5 is expressed and upregulated in PVRL models [39,41,42]. CXCR5 was expressed by PVRL as well as PCNSL and PTL lymphoma cells [29,41,43,44,47]. In systemic DLBCL, high expression of CXCR5 strongly correlated with secondary CNS infiltration [43]. Its ligand, CXCL13, was shown to be expressed by RPE and endothelial surface cells [41,42,44].

Our results show that CXCR5 was co-expressed on CD20-positive lymphoma cells in the PCNSL group. In contrast, no co-expression of CXCR5 was detected in the SCNSL group. Of all the receptors examined, CXCR5 was most frequently co-expressed. For that reason, we suspect that the CXCR5-CXCL13 axis could be particularly relevant for homing of lymphoma cells and pathogenesis of PVRL and PCNSL.

#### 3.2.3. CD20/CXCR7 Co-Expression

CXCR7 mRNA expression was associated with good prognosis in CXCR4 positive systemic DLBCL [49,50]. To our knowledge, it was not evaluated in PVRL so far. However, in a CXCR7 knockout mouse model of DLBCL, CXCR7 WT mice showed CNS infiltration as opposed to CXCR7 knockout mice, suggesting CXCR7 as an important receptor for CNS homing [49].

The results of our work show that in the choroid as well as in the retina and the ciliary body there were hardly any CD20-positive lymphoma cells which co-expressed the scavenger receptor CXCR7. However, we did not analyze CXCR7 mRNA expression. Thus, the significance of CXCR7 in homing of lymphoma cells to the brain and eye remains to be further evaluated.

#### 3.2.4. CD20/CD44 Co-Expression

CD44 has not yet been examined in PVRL but is expressed by lymphoma cells and vascular endothelium in PCNSL and PTL [11,46,51,52,53,54,55]. In PCNSL, lymphoma cells, which were often found perivascular, were CD44-positive [56]. Furthermore, CD44-positive B lymphocytes can infiltrate the white matter of the brain by binding to HA. This infiltration decreased when both the lymphocytes and the brain tissue were treated with either hyaluronidase or CD44 antibodies [51]. In PCNSL, expression of CD44 was associated with a shorter overall survival [56]. In PTL, CD44-expression seems to correlate with the late-stage of disease [55].

In our mouse model, CD44 was co-expressed only by a few CD20-positive lymphoma cells in the choroid and retina of the PCNSL group. Interestingly, CD44-positive cells were found in capillaries near CD20-positive lymphoma cells. We therefore assume that these cells may be CD44-positive monocytes or macrophages, which are involved in the extravasation of the lymphocytes by presenting chemokines, such as CCL4 and CCL5 [31,32,33,57]. It was shown that HA, the ligand of CD44 was expressed on vascular endothelium cells. Additionally, HA is also one of the main components of the extracellular matrix of the brain as well as the vitreous body of the eye [58]. Thus, CD44 positive lymphoma cells might migrate towards a HA gradient of the brain and eye. The CXCL12-CXCR4 axis can affect the expression and activation of CD44 [59]. In our study, the 15% proportion of CD20/CXCR4-positive cells in all CD20-positive lymphoma cells may have negatively impacted the expression of CD44 on lymphoma cells. CD44 remains an interesting candidate receptor for PCNSL and thus also for PVRL, which may well contribute to homing of lymphoma cells into the eye.

Together, our data for the first time demonstrate homing of human PCNSL, but not SCNSL, xenografts to the eye after heterotopic implantation and suggest that expression of chemokine receptors CXCR4, CXCR5 and CD44 may be involved in homing of lym-phoma cells into the eye.

### 3.3. Limitations

Although we used human CNSL xenografts, it must be emphasized that the data for this study using a PDX mouse model cannot be directly extrapolated to humans. In contrast to PVRL in humans [6], no lymphoma cells were present in the RPE in this model, even though a few lymphoma cells were found sub-RPE. This may result from specific representation of lymphoma cells by immunofluorescent staining of the mouse retina due to its slightly different morphology [60].

However, due to the scarcity of tumor material in PVRL, this mouse model creates the opportunity to expand human tumor material in vivo, allows us to specifically determine the exact localization of human CD20+ lymphoma cells in the eyes of PCNSL and SCNSL and is a valid model to investigate the significance of respective homing receptors for lymphoma manifestation in the CNS and eyes. Additionally, PDX mouse models unlike conventional xenograft mouse models preserve genetic heterogeneity over many passages. Therefore, a xenograft mouse model working with human lymphoma cells from patient samples offers a unique opportunity to study PVRL in vivo, creating the opportunity to identify mechanisms critical for homing of lymphoma cells that might be accessible for therapeutic intervention.

## 4. Materials and Methods

### 4.1. PDX Mouse Model

In this study we used a PDX PCNSL mouse model with a PVRL phenotype and compared it to a PDX SCNSL mouse model. These models were established by Isbell et al. (unpublished manuscript under review [36]). Tumor tissue from diagnostic stereotactic brain biopsies from patients with suspicion of PCNSL or SCNSL were implanted i.c. via the foramen postgleonidale into 4–6 weeks old recipient NSG mice (NOD NOD/Shi-scid/IL-2Rγnull; Charles River, France). DLBCL was detected in all patients, regardless of whether they were PCNSL or SCNSL. Monoclonality of lymphoma cells was demonstrated by FACS analysis of infiltrating lymphoma cells in the spleen of both PDX mouse models.

Secondary implantations were carried out after the depletion of mouse cells ((#130-104-694, Miltenyi Biotec, Bergisch Gladbach, Germany). A PDX was defined as established when stable growth was observed over at least 3 transplantation passages and regrowth from xenograft tumour tissue stored in liquid nitrogen was seen. Lymphoma cells from established PDX models were implanted i.s. into recipient NSG mice. Mice were euthanized after development of signs of disease. The eyes were enucleated and embedded into paraffin.

### 4.2. Primary and Secondary Antibodies

For indirect immunofluorescence staining, the following primary and secondary antibodies were used: Human CD20 (1:200, Abcam, Cambridge, UK, ab194970) [61], human/mouse CD44 (1:400, Thermo Fisher Scientific, Waltham, Massachusetts, USA, 14-0441-82) [62], mouse CXCR4 (1:25, Bio-Techne, Minneapolis, Minnesota, USA, MAB21651) [63], mouse/human CXCR5 (1:100, Abcam, ab133706) [64,65,66,67], mouse/human CXCR7 (1:200, Abcam, ab72100) [63], Donkey Anti-goat DyLight550 (1:200, Thermo Fisher Scientific, SA5-10087), Donkey anti-rat AlexaFluor 488 (1:200, Thermo Fisher Scientific, A21208) and Donkey anti-rabbit DyLight488 (1:200, Thermo Fisher Scientific, SA5-10038).

### 4.3. Double Indirect Immunofluorescence Staining

0.5 µm thick sections were cut from paraffin embedded eyes and mounted on slides. The slides were deparaffined using graded alcohol solutions. After boiling in citrate buffer (pH 6.0) and rinsing in phosphate buffered saline (PBS) the slides were incubated with 5% fetal bovine serum. The staining with primary antibodies against CD20 combined with primary antibodies against CXCR4 (1:25)/CXCR5 (1:100)/CXCR7 (1:200) or CD44 (1:400) took place during incubation overnight at 4 °C. The next day, the slides were washed in PBS and then stained with matching secondary antibodies for 1h at room temperature. For evaluation, the staining was imaged with immunofluorescence microscope (Leica DMI 6000 B, Leica, Germany). On each section, CD20-positive cells and co-expressing cells in the choroid, retina and ciliary body were counted using Fiji/ImageJ [68]. For evaluation with the immune reactive score (IRS), representative shots of the eyes were analyzed as explained below.

### 4.4. Cell Counting

To indicate a proportion, CD20-positive and co-expressing cells were counted manually by using the cell counter of Fiji/ImageJ [68]. The proportion then was calculated by dividing the number of CD20 and homing-receptor co-expressing cells by only CD20-positive cells.

### 4.5. Evaluation with the IRS

For every representative image of the choroid, retina, and ciliary body an IRS was determined. Firstly, the staining intensity of positive cells was estimated and scored as 0 = no reaction, 1 = weak reaction, 2 = moderate reaction and 3 = strong reaction. The percentage of positive cells was determined and scored. 0% positive cells were scored as 0, less than 10% as 1, 10–50% as 2, 51–80% as 3 and more than 80% as 4. The IRS was then calculated by multiplying the scores for staining intensity and percentage of positive cells [69,70]. We considered an IRS greater than 0 to be positive.

### 4.6. Statistical Analysis

Statistical analysis of ordinal scaled and not normally distributed IRS values was performed with the Mann–Whitney-U-Test using GraphPad Prism version 8.0.2 for Windows, GraphPad Software, San Diego, California, USA, www.graphpad.com (accessed 1 October 2022). A *p*-value less than 0.05 was considered statistically significant.

## 5. Conclusions

In this study, we were able to show that CD20-positive lymphoma cells in a PCNSL PDX homing mouse model with a PVRL phenotype co-express the chemokine receptors CXCR4, CXCR5 as well as CD44 in comparison to a SCNSL PDX mouse model. These receptors may therefore be involved in homing of B-lymphoma cells into the eyes in PVRL. The expression patterns of these homing receptors and their ligands CXCL12, CXCL13 and HA should be further researched, in knock-out models and in knock-out DLBCL cell lines, for example. Furthermore, downstream investigations of homing receptors are of particular interest as well as alteration of these chemokines in the process of homing within this mouse model. Chemokines and the receptors themselves may help us to understand this disease better and therefore might introduce possible new targets for therapeutic approaches for PVRL in the future.

## Data Availability

Not applicable.

## Supplementary Materials

The following supporting information can be downloaded at: https://www.mdpi.com/article/10.3390/ijms231911757/s1, Figure S1: Bar charts of proportions of co-expressing cells.
