# Supplementary figures and images for: CXCR4, CXCR5 and CD44 May Be Involved in Homing of Lymphoma Cells into the Eye in a Patient Derived Xenograft Homing Mouse Model for Primary Vitreoretinal Lymphoma
Source: Int J Mol Sci. 2022 Oct 4;23(19):11757. doi: 10.3390/ijms231911757 (PMC9569795; doi:10.3390/ijms231911757)

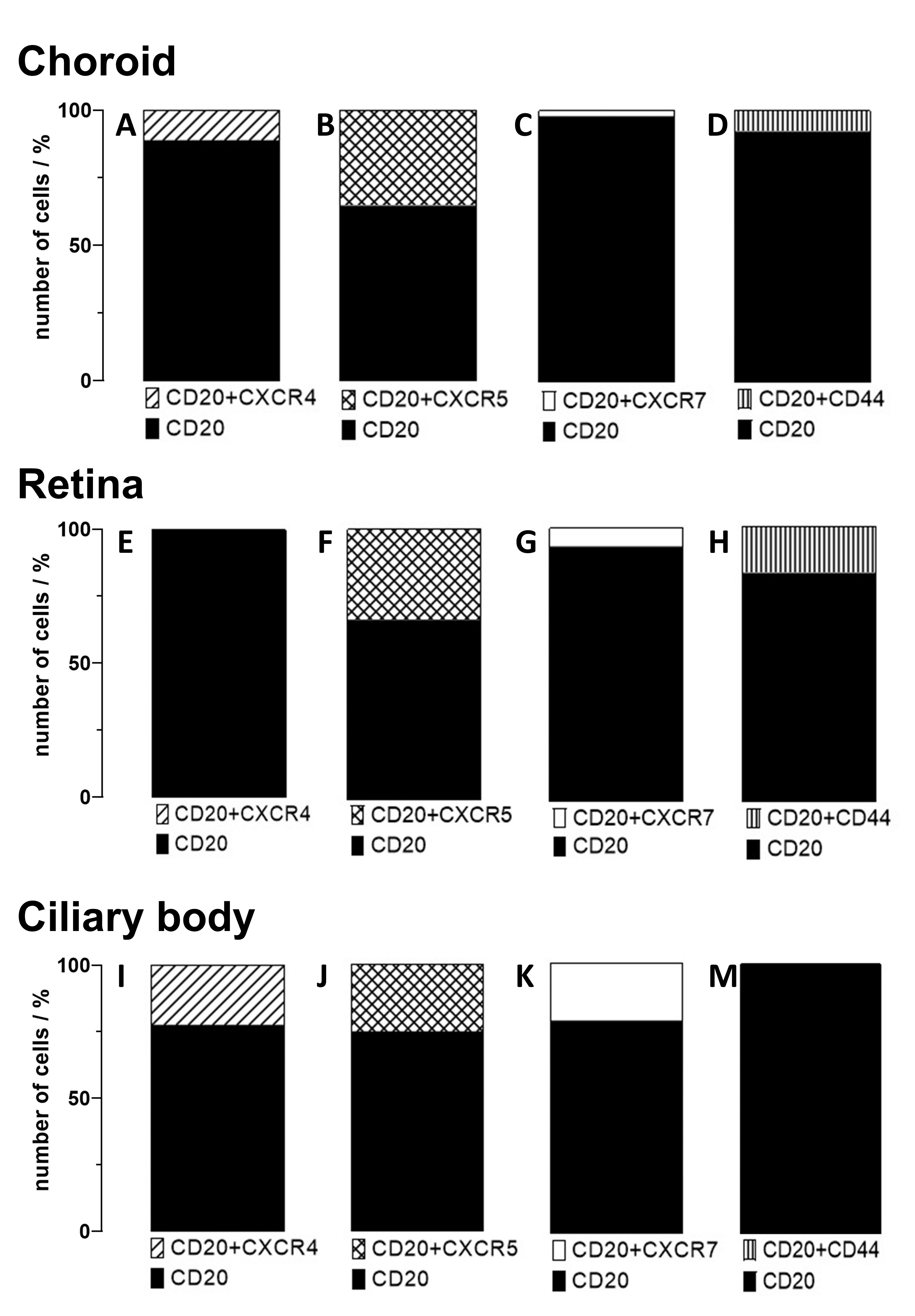

Supplement: Supplementary file 1 [file ijms-23-11757-s001.zip › Supplementary/Figure S1.jpg]
